# IgG replacement in multiple myeloma

**DOI:** 10.1038/s41408-024-01107-6

**Published:** 2024-07-25

**Authors:** Alex Wonnaparhown, Talal Hilal, Jacqueline Squire, Catherine Freeman, Rafael Fonseca

**Affiliations:** 1 Division of Allergy, Asthma, and Clinical Immunology, Mayo Clinic, Phoenix, AZ USA; 2 Division of Hematology and Medical Oncology, Mayo Clinic, Phoenix, AZ USA; 3 Division of Allergy, Asthma, and Clinical Immunology, Mayo Clinic, Jacksonville, FL USA

**Keywords:** Myeloma, Immunological deficiency syndromes

## Abstract

T cell engagers (TCE) such as chimeric antigen receptor (CAR) T cell therapy and bispecific antibodies (BiAbs) for the treatment of multiple myeloma (MM) have significantly improved clinical outcomes, but have also raised awareness for ensuing post-treatment secondary immunodeficiency and hypogammaglobulinemia (HG). As patients with MM live longer, recurrent infections become a significant component of therapy-associated morbidity and mortality. Treatment of HG with immunoglobulin G replacement therapy (IgG-RT) has been a mainstay of the primary immunodeficiency (PI) world, and extrapolation to MM has recently started to show promising clinical outcomes. However, IgG-RT initiation, dosing, route, timing, monitoring, and management in MM has not been standardized in the setting of TCE. Progress in MM treatment will involve greater recognition and screening of underlying secondary immunodeficiency, identification of risk-stratification markers, optimizing IgG-RT management, and implementing other approaches to decrease the risk of infection. In this review, we summarize infection risk, risk of HG, and management strategies for IgG-RT in patients with relapsed MM after TCE.

## Introduction

Due to the advent of new therapies, survival has significantly improved for patients with MM. Many of these therapies also inadvertently target other cellular elements of the patients’ adaptive immune system such as late B cells, normal plasma cells, and even T cells. Historically, triple-class refractory disease (progression after immunomodulatory agents, proteasome inhibitors, and anti-CD38 antibodies) has been associated with poor patient outcomes and median overall survival of about 9 months [1, 2]. The advent of TCE has resulted in improved outcomes for this patient population, now resulting in median overall survival of greater than 2–3 years [3]. As patients with MM live longer, secondary immunodeficiency and recurrent infections become significant obstacles associated with morbidity and mortality [4]. In this review, we summarize mechanisms of immunodeficiency, HG, and management with IgG-RT in patients with MM with a particular focus on post-TCE.

## Immunodeficiency in MM

**Fig. 1:**
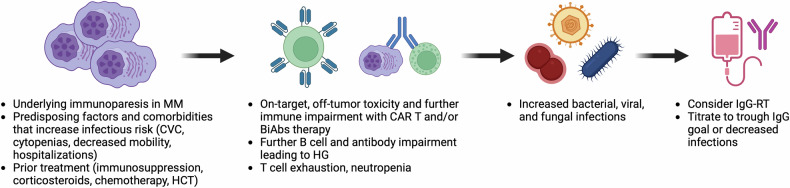
Mechanism of immune dysregulation in MM. Abnormal expansion of malignant plasma cells in MM leads to immunoparesis, impaired immunity, and HG. Additional treatment and other comorbidities further impair other arms of the immune system and lead to increased infections. Initiation of IgG-RT can be considered to reduce severe bacterial infections.

The underlying immunodeficiency seen in MM reflects disruption of both adaptive and innate immunity (Fig. 1). HG occurs in patients with untreated myeloma and those receiving myeloma-directed therapy and has important management implications. The 2022 American Academy of Allergy, Asthma, and Immunology (AAAAI) Work Group Report defines HG as an IgG level <700 mg/dL [5]. The cutoff for HG in various other studies has ranged from 500–700 mg/dL and has also been stratified into mild (IgG 400–599 mg/dL), moderate (IgG 200–399 mg/dL), and severe (IgG <200 mg/dL) HG [6–8].

Abnormal expansion of malignant plasma cells in MM leads to an impaired polyclonal humoral response and subsequent HG [9]. This decline in functional immunoglobulin, a term defined as immunoparesis, is found in over 90% of patients with newly diagnosed MM and is considered by most clinicians to be associated with increased risk of bacterial infections [10]. In patients with IgG MM, whose IgG serum concentrations may be in the normal range but is comprised of monoclonal and non-functional antibodies, identification of functional HG should be considered. Immunoparesis is not restricted to MM and can also be found in 25–40% of patients with monoclonal gammopathy of undetermined significance (MGUS), 52% of smoldering MM, and 75% of MM in remission [5, 10]. Although not typically thought of as an immunodeficiency state, a population-based study in Sweden found that MGUS patients had a 2.1-fold increased risk of developing any infection compared to controls, although the average number of infections per patient was 0.34 compared to 0.17 [11]. At 10 years, MGUS patients also had an increased risk of pneumonia, osteomyelitis, sepsis, pyelonephritis, cellulitis, endocarditis, and meningitis.

Further associated immune defects in MM include decreased CD19^+^ B cells, decreased CD4^+^ T cells, decreased CD8^+^ T cells, inverted CD4:CD8 ratio, dysfunctional dendritic cells, increased regulatory T cells, and diminished NK cell function [12, 13]. This resulting immunodeficiency in MM leads to an increased risk for infection, highlighted in a population-based study conducted in Sweden that showed MM patients had a 7 and 10-fold increased risk for developing bacterial and viral infections respectively compared to controls [14].

## Conventional therapies (e.g., triplets, quadruplets)

Patients with newly diagnosed MM are vulnerable to infections and early mortality. Population-level data reported an early mortality rate between 10% within 60 days to 28% within 1 year [15, 16]. The most frequent causes of death were infections (45%), followed by renal failure and vascular events. These infections are often driven by higher rates of neutropenia associated with induction regimens. In both the frontline and relapsed/refractory settings, daratumumab, an anti-CD38 monoclonal antibody, was associated with higher rates of infection overall (RR 1.27; 95% CI 1.17–1.37), severe infections (RR 1.27; 95% CI 1.14–1.41), and pneumonia (RR 1.39; 95% CI 1.12–1.72) [17]. In addition to neutropenia, daratumumab is associated with depletion of NK cells, which results in higher rates of viral reactivation (e.g., herpes zoster, varicella zoster) [18]. Immunomodulatory drugs (e.g., lenalidomide, pomalidomide) also result in increased risk of infection due to higher rates of neutropenia. The types of infections are typically pneumonia and urinary tract infections. Antimicrobial prophylaxis is often implemented in this setting, while IgG-RT is seldom used in routine practice.

## CAR T cell therapy

The development of CAR T cell therapy targeting B cell maturation antigen (BCMA) and other epitopes such as G-protein coupled receptor family C group 5 member D (GPRC5D) has drastically improved the ability to control advanced MM, but with a consequent risk of increasing infections. Idecabtagene vicleucel (ide-cel) targeting BCMA was approved in 2021 for MM refractory to 4 or more lines of therapy [19]. One year later, ciltacabtagene autoleucel (cilta-cel) was approved in a similar patient population [20]. In clinical trials, these therapies showed high response rates (73–97%) and greater durability of response compared to standard of care. However, 21% of patients developed HG in the ide-cel trial and a following study showed that up to 76% of patients developed HG 1-year post-BCMA CAR T cell therapy [19, 21]. In the CARTITUDE-1 trial, among 97 patients treated with cilta-cel, 94% developed HG [20]. The incidence of infections after BCMA CAR T cell therapy is between 37–53% and most infections occur in the first 100 days [21, 22]. After day 100 and within the first year after CAR T cell therapy, respiratory infections predominate, and IgG <400 mg/dL has been associated with a higher risk of late respiratory infections [22]. In addition to post-treatment infection risk, most patients undergoing CAR T cell therapy also have preexisting humoral immunodeficiency with low peripheral CD19^+^ B cells and HG related to prior treatment—up to 42% of patients in one analysis had an IgG <300 mg/dL prior to receiving lymphodepleting chemotherapy [21].

When using CAR T cell therapy in earlier lines of treatment, these infectious risks persist. The phase 3 KarMMa-3 trial comparing ide-cel versus standard of care therapy after 2–4 prior lines of therapy showed superior progression-free survival responses [23]. Adverse events in the ide-cel group included neutropenia (78%), lymphopenia (73%), and infection (58%). Infections occurred during or after ide-cel infusion in 82%, and the most common infections included upper respiratory tract infection (12%) and pneumonia (10%). Additional data suggests that grade 3/4 infections remain consistent over time with ide-cel, but bacterial infections are more common within 3 months of treatment [24]. The phase 3 CARTITUDE-4 trial comparing cilta-cel versus standard of care therapy after 1–3 prior lines of therapy also showed decreased disease progression and death in refractory MM patients [25]. Adverse events in the cilta-cel group included neutropenia (89.9%), lymphopenia (22.1%), and infection (62%). Upper respiratory tract infections were seen in 18.8%, pneumonia and bronchitis in 9.1%. HG was reported in 90.9% and IVIG was administered in 65.9% of the cilta-cel group.

CAR T cell therapy can have profound and long-lasting effects on immunity. Studies on CD19-targeted CAR T cell therapy have shown that significant endogenous B cell depletion occurs in the first 28 days and only approximately 21% of patients recover B cell numbers by day 90 [26]. After tisagenlecleucel treatment, median time to sustained B cell recovery was 6.7 months. Multiple other studies have also shown long-term persistence of CAR T cells that can last months to years after infusion, resulting in prolonged B cell aplasia that can persist for a mean of 571 days [27, 28]. A study of two patients treated with CD19 CAR T cells for CLL revealed detectable CAR T cells more than 10 years after infusion [29].

Although kinetics have not yet been extensively studied in BCMA-targeted CAR T cell therapy, continued diminished CD19^+^ B cells and switched memory B cells have been seen at a median of 20 months post-therapy [30]. BCMA-targeted CAR T therapy has also been associated with impaired vaccine responses [27]. One study investigated the kinetics of B cell, normal plasma cell, and immunoglobulin recovery in 40 patients who achieved response after anti-BCMA CAR T cell therapy [31]. The median duration of B cell aplasia was 70 days (range 23–270). Normal plasma cells in the bone marrow were first re-detected at a median of 212 days. Moreover, virus-specific IgG levels decreased over time and 57% of patients had a total of 44 infection events. Overall, these results suggest a profound and lasting humoral immunodeficiency after CAR T cell therapy.

## Bispecific antibody therapy

BiAbs target both CD3 expressed on the surface of T cells and a tumor antigen such as BCMA, expressed on the surface of MM cells, thus mediating T cell activation and subsequent cell death [7]. There are several antigens that BiAbs have been developed to target, which include BCMA (teclistamab, elranatamab, alnuctamab, and linvoseltamab), GPRC5D (talquetamab), and FcRH5 (cevostamab). While these therapeutic targets are preferentially expressed in plasma cells (normal and malignant), they are also expressed in other late B cells, creating an array of humoral immunodeficiency beyond the mere depletion of normal plasma cells. These therapies also have the potential to impair immunity and vaccine antibody responses. In contrast to CAR T cell therapy, BiAb treatments are administered on an ongoing basis, sometimes indefinitely, which raises the concern for prolonged immunosuppression with lymphocyte depletion (both B and T cells) and T cell exhaustion. A study of 37 patients on IgG-RT and treated with BCMA-targeted BiAbs found an infection rate of 3.3 per patient-year, and 26 of these infections were grade 3–5 [7]. Most infections involved the respiratory tract (58%), and etiologies of infections were viral (46%), bacterial (43%), and fungal (11%).

The first FDA-approved BiAb was teclistamab in 2022. In the MajesTEC-1 trial, 44.8% of the 165 patients had grade 3/4 infections within the median follow-up period of 14.1 months and HG (by report and/or IgG <500 mg/dL) was seen in 74.5% of patients [8]. Infections included pneumonia (18.2%), COVID-19 (17.6%), bronchitis (13.3%), upper respiratory tract infection (10.9%), and *Pneumocystis jirovecii* pneumonia (PJP) (3.6%). Elranatamab gained approval in 2023 based on results from the phase 2 MagnetisMM-3 trial [32]. In total, 69.9% of the 123 patients had infections at a median follow-up of 14.7 months. These included grade 3/4 infections in 40% (including 6 cases of PJP) and fatal infections in 6.5%. HG (IgG <400 mg/dL) was seen in 75.5%, and IgG-RT was given to 43% of patients. In the talquetamab registration trial, infections occurred in 47% of patients, including 7% grade 3/4 infections [33, 34]. HG (IgG <500 mg/dL) occurred in 87% of patients who received the 405 μg dose and in 71% of those who received the 800 μg dose. Results from the Phase1b MonumenTAL-2 study looking at talquetamab and pomalidomide combination therapy showed that 80% had any grade of infection, including 8 of 35 with pneumonia [35]. CD19^+^ B cells did not decrease during treatment, however 77.1% of patients had post-treatment IgG <400 mg/dL and 34.3% received IVIG.

Because of different patterns of surface expression for each BiAbs, the risk of HG and infections may not be universal. This difference could be due to the construct potency or because of the distribution of these surface markers in other immune system cells. Targeting GPRC5D has been thought to be associated with a lower risk of infection. In a cohort of 29 patients receiving GPRC5D-targeting BiAbs and 200 patients receiving BCMA-targeting BiAbs, the cumulative incidence of infection in the anti-GPRC5D group was 53% compared to 73% in the anti-BCMA cohort [36]. The range of infections observed with the use of BiAbs seems to be a consequence of combined humoral and T cell dysfunction, and the constant recruitment of T cells is believed to lead to their exhaustion.

## Additional immunosuppressive concerns with TCE

**Fig. 2:**
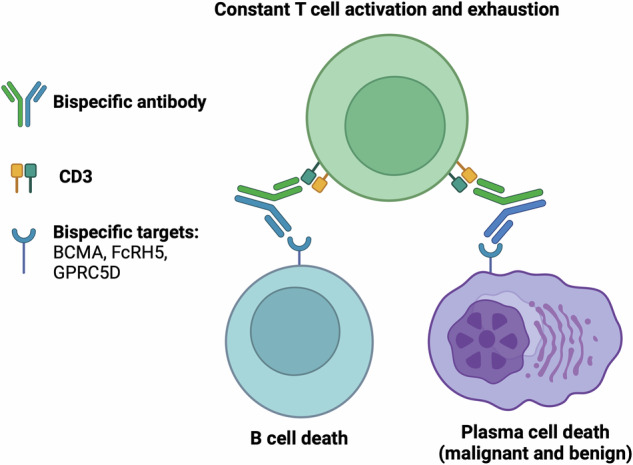
T cell exhaustion. TCE therapy can lead to T cell exhaustion, characterized by progressive loss of effector function and reduced proliferative capacity. The mechanism underlying this process is thought to be related to persistent antigen stimulation and immunosuppressive tumor microenvironment.

In addition to HG, other risk factors for infection exist in patients treated with BCMA-targeted cellular therapies. The prevalence of grade 3 or higher neutropenia ranges from 13–78% in patients treated with BCMA-targeting monotherapy and/or combination therapy [37]. Furthermore, T cell exhaustion is a dysfunctional state of T cells characterized by progressive loss of effector function and reduced proliferative capacity [38]. This phenomenon is seen after CAR T cell therapy and is a major limitation to treatment efficacy. The mechanisms underlying T cell exhaustion are complex but likely related to persistent antigen stimulation and the immunosuppressive tumor microenvironment (especially in solid tumors) (Fig. 2). Infections associated with T cell depletion or exhaustion include PJP, CMV and invasive aspergillosis, all of which have been reported after both CAR T cell therapy and BiAb treatment [37].

## IgG-RT

IgG-RT is available as intravenous (IV) or subcutaneous (SC) and has been instrumental in treatment and infection prophylaxis in PI and various other conditions. Intravenous immunoglobulin (IVIG) is currently FDA-approved for PI, B cell chronic lymphocytic leukemia, Kawasaki disease, bone marrow transplantation, pediatric HIV, idiopathic thrombocytopenic purpura, chronic inflammatory demyelinating polyneuropathy, and multifocal motor neuropathy [39]. IgG-RT use has been shown to be most beneficial in reducing severe bacterial infections in severe antibody deficiency diseases and agammaglobulinemia. The role of IgG-RT in preventing viral infections is less clear, although may have evidence in the treatment of CMV pneumonitis, rotaviral enterocolitis, enteroviral meningoencephalitis, RSV, and chronic parvovirus B19 [39]. A 2022 systematic review and meta-analysis of randomized controlled trials did not show improved clinical outcomes when IVIG was used to treat hospitalized COVID-19 patients, however the presence of donor-derived specific SARS-CoV-2 antibodies in the infused IVIG products was unknown [40]. Studies in agammaglobulinemia have shown that IgG trough levels >800 mg/dL prevent serious bacterial illnesses and IgG trough levels >1000 mg/dL decrease episodes of pneumonia. Meta-analyses of CLL, MM, and HCT clinical trials have not shown reduced mortality with IgG-RT [41]. However, many of the included studies were older, had short follow-up duration, and were limited by heterogeneity of the various hematologic malignancies. More recent studies show promising benefit with IgG-RT after CAR T and bispecific therapy.

## Effectiveness of IgG-RT in reducing infections

Studies assessing the benefit of IgG-RT in MM have shown conflicting data. However, many studies have been retrospective and challenging to standardize with the heterogenous and rapidly changing field of MM therapy. Initial studies in 1994 and 1995 showed significantly reduced life-threatening infections with IVIG use in stable-phase MM [42, 43]. In contrast, a systematic review and meta-analysis in 2009 looking at CLL and MM showed that IVIG decreased major infections but did not show survival benefit [44]. A retrospective study of 162 MM patients in 2015 also did not show a significant difference in rates of infection with IVIG use after autologous transplantation [9]. Twelve doses of 500 mg/kg of IVIG were given post-transplant by physician discretion, but IgG trough levels were not monitored. A retrospective study of 266 patients after autologous transplant for MM also did not find benefit with IVIG dosed at 400 mg/kg [45]. Inclusion criteria included patients with a total combined IgG, IgA, and IgM of less than 8 g/L. However, IVIG dosing was not standardized, only 19.2% were on regularly scheduled IVIG infusions, IgG troughs were not reported, and all but 6 patients had a central venous catheter (CVC).

More recently, a 2023 retrospective study in MM patients after BiAb therapy showed universal profound HG in responders and a significant decrease in grade 3–5 infections while on IVIG [7]. The median time to severe HG was 2.9 months. IgA and IgM became undetectable by the second month and remained undetectable for the duration of therapy. 92% of responders received IVIG for a median of 10 doses, typically given at 400 mg/kg every 4 weeks regardless of the IgG level. The same research group also showed significant reduction in all infectious events and grade 3 or higher infectious events with IVIG in non-progressive disease MM and during daratumumab treatment [7, 46]. Newly published clinical trial data showed that teclistamab significantly reduced peripheral B cells, plasma cells, polyclonal immunoglobulins, and vaccine responses that did not recover while patients had ongoing treatment [47]. IVIG was given every 4 weeks outside of the CRS window starting at 10 g for IgG levels <400 mg/dL to achieve a goal IgG of >400 mg/dL and showed a substantial decrease in serious infections (1.36 per patient-year in the observation group, compared to 0.12 per patient-year in the IVIG group).

## Indications for initiation of IgG-RT

Clear guidelines on IgG-RT initiation in MM have not been outlined. Starting IgG-RT in MM depends on multiple factors including comorbidities, prior MM treatment, ongoing clinical condition, and shared decision-making discussing risks and benefits with the patient. Furthermore, the decision to initiate IVIG in MM can be challenging if there is a paraprotein that is falsely elevating or normalizing the IgG level. For IgG myeloma, one approach is to subtract the M-spike from the total IgG to estimate polyclonal functional IgG [7]. Although not prospectively validated in MM, this strategy parallels the clinical picture in various PI, such as activated PI3K delta syndrome, which can present with normal or elevated IgG, but with poor vaccine responses and elevated risk of infection.

The 2022 AAAAI Work Group Report outlines general considerations for starting IgG-RT in secondary HG (Table 1) [5]. In patients with a history of recurrent infections, IgG-RT could be considered for (1) IgG <400 mg/dL or (2) IgG <700 mg/dL with additional low IgA and/or IgM and impaired vaccine responses. In patients without recurrent infection, IgG-RT should still be considered for an IgG <150 mg/dL. In contrast, hematopoietic cell transplantation (HCT) guidelines do not recommend routine administration of IVIG within the first 100 days after transplantation to prevent bacterial infections [48]. However, IVIG could be considered with a low IgG < 400 mg/dL with the goal to maintain an IgG level of >400 mg/dL. In 2018, the American Society for Blood and Marrow Transplantation (ASBMT) also recommended against IgG-RT without a history of recurrent infections regardless of IgG level in transplanted patients [49]. Without definitive guidelines, another strategy in B cell malignancies is to check serum IgG prior to and within 3 months post-CAR T therapy and consider IgG-RT if the IgG is ≤400 mg/dL [26, 50, 51]. The significant infection risks of TCE may even warrant monthly IgG monitoring post-treatment. Extension of immunoglobulin monitoring could be considered for up to 6 months after therapy and then twice yearly thereafter [50, 52].

**Table 1:** When to initiate IgG-RT.

2022 AAAAI Work Group Report for Secondary HG	• IgG < 400 mg/dL with recurrent infections • IgG < 700 mg/dL with recurrent infections, in addition to low IgA or IgM, and impaired vaccine responses • IgG < 150 mg/dL
HCT guidelines	• Not routinely recommended, but can be considered if IgG <400 mg/dL
ASBMT	• Recommend against IgG-RT if no history of recurrent infection, regardless of IgG level
Hill et al.	• Check serum IgG prior to and within 3 months post-CAR T cell therapy and consider if IgG is ≤400 mg/dL • IgG 400–600 mg/dL with serious recurrent infections • IgG > 600 mg/dL with impaired vaccine responses

## Monitoring

In general, immunoglobulin levels (IgG, IgA, and IgM), vaccine responses, and B and T lymphocyte subsets should be obtained at baseline prior to initiation of IgG-RT (Table 2) [5]. An IgG trough level is then typically obtained 3 months after initiation of IVIG therapy (prior to the fourth infusion), to assess response to initial dosing and to guide IgG-RT dose adjustment (Table 3). However, there may be benefit to monitoring IgG levels monthly and enacting a more aggressive approach to quickly reaching therapeutic IgG trough levels. For patients with a stable IgG at goal, IgG trough monitoring can then be spaced out to every 6–12 months. Timing of IgG collection on stable treatment with weekly SCIG is less important, as pharmacokinetic studies have demonstrated a near steady-state IgG level between infusions [53]. If the underlying immunosuppressive treatment is discontinued, immunoglobulins (IgG, IgM, and IgA) and B cell subsets (CD19^+^ and CD20^+^) can be monitored to assess for improving B cell function [26]. Once IgG-RT is discontinued, re-evaluation of IgG levels to assess for endogenous recovery can be performed in approximately 3–4 months, accounting for the 21-day half-life of IVIG and 4–5 total half-lives to clear exogenous IgG [5].

**Table 2:** Screening considerations.

• Baseline immunoglobulins (IgG, IgM, and IgA), vaccine responses (tetanus, diphtheria, *Pneumococcus*), and B and T lymphocyte subsets prior to CAR T
• Check IgG within 3 months post-CAR T (consider checking monthly for high-risk patients)
• Monitor IgG for 6 months after CAR T and then twice yearly thereafter

**Table 3:** Monitoring while on IgG-RT.

• IgG trough levels (before IVIG infusion) every 2–4 months until at goal
• Timing of IgG monitoring matters less in SCIG steady-state
• IgG trough monitoring every 6–12 months when at goal
• Intermittent IgA and IgM monitoring
• CBC w/ differential, renal function, hepatic enzymes every 6–12 months
• Serum viscosity if paraproteinemia present
• Close monitoring for thromboembolic risk factors

The AAAAI PI practice parameter recommends routine monitoring of blood cell counts and serum chemistry every 6–12 months while on IgG-RT to monitor for cytopenia, hemolysis, hepatitis, and renal disease [54]. Renal dysfunction, cytopenia, and thrombosis are especially important to monitor in patients with MM receiving IgG-RT [55]. Patients with additional cardiovascular risk factors, such as paraproteins, should also be closely monitored for clinical thromboembolic manifestations and with blood viscosity assessment [26]. For those at higher cardiovascular risk, IgG-RT should be administered slowly or subcutaneously [54].

## Optimal IgG trough goal

IgG-RT management strategies differ in hematologic malignancy when compared to PI and consist of either intermittent or scheduled dosing (Table 4). Primary hematologic and initial PI literature often recommend aiming for a trough level of at least 400–500 mg/dL [5, 39, 48, 56]. One strategy is intermittent IVIG dosing to maintain a minimal trough level of 400 mg/dL for the first 3 months after CAR T therapy [41]. In contrast, typical IgG-RT dosing in PI consists of routine monthly to weekly dosing with dose adjustments to target an IgG trough level of 800 mg/dL and a higher goal of 1000 mg/dL if the patient has recurrent pulmonary infections [5, 39]. More recent studies in PI literature support that a higher trough IgG, at least 700–850 mg/dL, results in less frequent infections and better patient outcomes [39]. The AAAAI Work Group Report recommends an initial IgG goal of 800 mg/dL [5]. A meta-analysis demonstrated a progressive decline in incidence of pneumonia with increasing trough IgG levels [56]. Along this line, the concept of an individual “biologic” trough level for each patient has been accepted within the PI community with the idea to titrate IgG-RT dosing based on infection history instead of using a fixed weight-based dose [26, 57]. Therefore, if objective severe infections continue despite normal IgG level on IgG-RT, increasing the dose of IgG-RT can still be considered. Since many studies in CLL, HSCT, and MM have adopted a fixed single weight-based Ig-RT dose for patient cohorts, higher doses may be needed to reduce morbidity and mortality in these cohorts. Increasing IgG-RT dosing based on infectious history should be weighed against the risks of supratherapeutic IgG levels in the setting of malignancy. For patients who experience side-effects and/or volume concerns, a lower dose given more frequently with pre-medications can be considered.

**Table 4:** IgG-RT dosing and IgG goal.

Kampouri et al.	• IVIG 400 mg/kg every 3–4 weeks or SCIG 100–200 mg/kg/week • IgG trough >400 mg/dL for first 3 months after CAR T therapy
2022 AAAAI Work Group Report	• IVIG 400–600 mg/kg/month or SCIG 100–200 mg/kg/week • IgG trough >800 mg/dL (>1000 mg/dL if recurrent pulmonary infections) • Titrate to clinical improvement
Hill et al., Bonagura et al.	• IVIG 400–800 mg/kg every 3–4 weeks or SCIG 100–200 mg/kg/week • IgG trough >400 mg/dL • Titrate to clinical improvement

## Subcutaneous IgG-RT (SCIG)

Recent studies suggest that SCIG maintains more stable IgG trough levels, reduces infections, and has lower adverse reactions than IVIG. A systematic review and meta-analysis in 2019 looked at 24 observational studies in PI and assessed that SCIG was able to achieve a 75.43 mg/dL higher mean trough level than IVIG, with each 100 mg/dL increase in IgG trough in SCIG associated with reduced rates of pneumonia [58]. Higher IgG troughs seen in SCIG may be explained by favorable pharmacokinetics of weekly dosing compared to monthly dosing. However, limitations of these studies are that many were developed by pharmaceutical companies with potentially biased data.

Although most studies to date evaluated IVIG in MM, one randomized controlled trial of 46 MM patients on SCIG showed a reduction in annual severe infections, good safety profile, only local skin adverse events, better quality of life, and better cost-effectiveness [59]. Patients in this study had an IgG <500 mg/dL and were infection-free at trial entry. A dose of 400–800 mg/kg/month divided into weekly doses was used and adjusted with a goal to maintain IgG >500 mg/dL. Zero patients in the SCIG arm developed sepsis, bacterial pneumonia, or acute sinusitis, compared to 24, 18, and 5 in the control arm respectively. Three patients had a non-anaphylactic Grade 3/4 reaction prompting SCIG discontinuation. An additional retrospective study also found benefit in using at least 12 weeks of SCIG in secondary antibody deficiency, including in MM, CLL, and non-Hodgkin lymphoma [60]. These studies suggest a promising route with SCIG that may benefit patients by reducing side-effects, in addition to improving ease of home administration.

## Risks of IgG-RT

Overall, mild systemic flu-like adverse effects may affect up to 25% of patients receiving IVIG, but are minimal with SCIG [61]. While few patients experience severe side-effects with IgG-RT in general, certain additional risks should be recognized when starting IgG-RT especially in the setting of MM, including acute renal failure and thromboembolism. Acute renal failure is a rare complication of intravenous immunoglobulin use, with an estimated incidence of less than 1% [62]. Tubulo-interstitial nephropathy has been associated with sucrose in IVIG preparations, which has now been removed from most current immunoglobulin products. However, renal injury has also been reported with maltose and glucose containing products.

Since 2013, the FDA has required a boxed warning on all non-specific immune globulin products, regardless of route of administration, regarding the associated risk of thromboembolic adverse events (TEEs) [63]. This warning also details potential risk factors, many of which are relevant in MM, including advanced age, prolonged immobilization, hypercoagulable conditions, indwelling vascular catheters, hyperviscosity, and cardiovascular risk factors. A retrospective cohort study of 14,944 patients showed a 15.6 per 1000 persons rate of same-day thrombotic event diagnosis [64]. A subsequent systematic review and meta-analysis of 28 randomized controlled trials, including 2318 IVIG-treated participants, evaluated the relationship between IVIG treatment and clinically serious TEE risk [65]. No increased TEE risk among patients who received IVIG compared with placebo or no treatment was identified. No statistically significant increased risk was found when arterial and venous TEEs were analyzed as separate endpoints either. Of note, IVIG use for a variety of indications was included and patients with immunodeficiency were underrepresented in this study analysis. More relevant results were noted when TEEs were specifically evaluated in patients with CLL and MM treated with IVIG [63]. A total of 2724 IVIG users (649 with MM) were compared to 8035 non-users. Acute myocardial infarction and ischemic stroke risk were three times higher during days 0 to 1 following IVIG treatment. In patients treated with IVIG for 1 year, the estimated absolute risk increase of a severe TEE was approximately 1%. No statistically significant increase in risk of venous TEE was noted.

The specific mechanism for increased thrombotic risk in MM is not known and there are many potential confounding variables from hematologic malignancy treatment [66, 67]. Mechanisms for thrombosis may include an increase in blood viscosity, erythrocyte aggregation, platelet activation, arterial vasospasm, and residual coagulation factors [63]. A population-based study in Sweden found that risks for thrombosis were higher closer to initial diagnosis in both MM and MGUS compared to healthy-matched controls, however M-protein concentration did not correlate with thrombotic risk in MGUS patients [67].

## Discontinuing IgG-RT

Without accurate biomarkers of infection risk, the duration of IgG-RT relies on shared decision-making and risk-benefit discussions with the patient (Table 5). After starting IgG-RT, the 2022 AAAAI Work Group Report recommends re-assessment every 6–12 months and to consider pausing IgG-RT 9–12 months after B cell-depleting therapy has been discontinued [5]. If IgG-RT is paused, immunoglobulins and vaccine responses can be rechecked 3–4 months later to re-evaluate if the patient meets criteria for re-initiation of IgG-RT. Another strategy has been to see if patients can maintain adequate IgG levels ≥400 mg/dL for three consecutive months off IgG-RT without evidence of recurrent bacterial infections [26, 41]. Recovery of peripheral CD19^+^ and CD20^+^ B cells, along with normalization of IgG, IgM, and IgA can also be monitored to support return of B cell function [26].

**Table 5:** Considerations for discontinuing IgG-RT.

• Consider pausing IgG-RT 9–12 months after discontinuation of B cell-depleting therapy
• Monitor for endogenous recovery of CD19^+^ B cells, CD20^+^ B cells, IgG, IgM, and IgA
• Able to maintain IgG ≥400 mg/dL for three consecutive months off IgG-RT without evidence of recurrent bacterial infections
• Protective vaccine responses checked 3–4 months after IgG-RT discontinuation

## Future directions

The heterogenous nature of current MM therapy and various confounding factors makes infectious risk-stratification challenging. Closer attention to baseline immune markers, such as immunoglobulins and lymphocyte subsets, may be key in identifying clinical and biologic changes with MM treatment that are associated with infectious outcomes. Ideally, baseline immune function would be obtained at a healthy state years before signs and manifestation of disease. As technology improves, helpful biomarkers may be discovered in the realm of genomics, B cell receptor repertoire, unique B and T lymphocyte subsets, microbiome, proteomics, and metabolomics.

The increase in infection risk alongside novel therapies in MM reflects multifactorial suppressive effects on the immune system [14]. Current strategies to prevent infections include prophylactic antibiotics, vaccination, and IgG-RT. Future studies investigating benefit from IgG-RT in MM should be designed in the context of already known benefits from IgG-RT, such as reduction in serious bacterial infections and pneumonia. Studies should also pay special attention to other infectious risk factors, such as CVC placement and hospitalizations, which may not be mitigated with IgG-RT.

IgG-RT management strategies widely vary between clinicians, and studies assessing IgG-RT use in MM have not implemented standardized management algorithms. An ideal future direction would involve prospective randomized, double-blind placebo-controlled trials assessing a standardized population to determine optimal initiation, dosing, monitoring parameters, and routes of administration. Future collaboration combining hematologic malignancy expertise with Allergy/Immunology immunodeficiency and IgG-RT expertise will benefit outcomes. Assessing appropriate timing of IgG-RT therapy will also be critical, especially if administered concomitantly with monoclonal antibodies and other TCE. Potential concerns include additive complications with CRS and competition for antibody binding sites. However, adding an extra infusion day may further increase healthcare-related costs and scheduling burden. These concerns may potentially be alleviated by utilizing the benefits of steady-state dosing and reduced side-effects with SCIG instead of IVIG.

## Summary

**Fig. 3:**
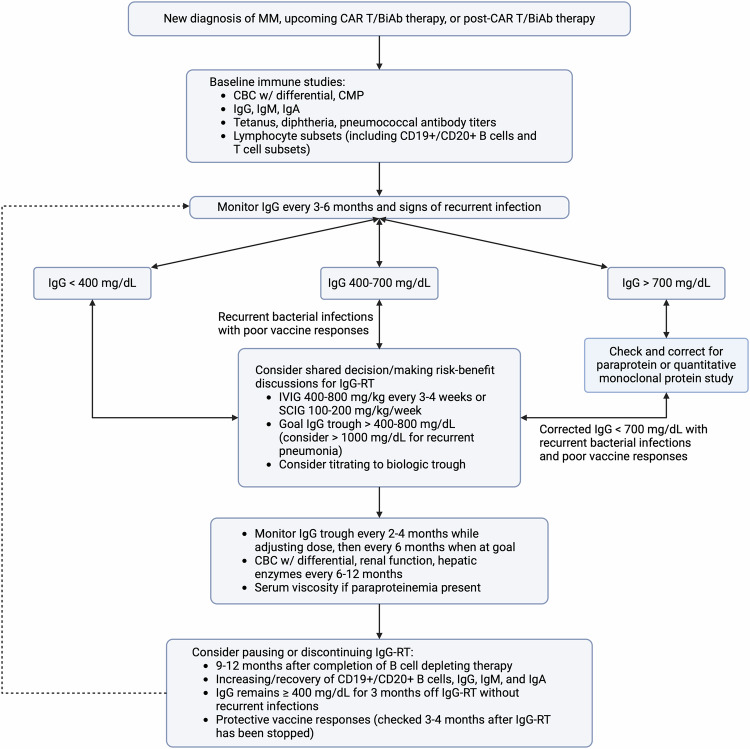
Framework for initiating and managing IgG-RT in MM. Review of both PI and hematologic malignancy literature supports the importance of obtaining baseline immune evaluation prior to immunosuppressive treatment and patients receiving immunosuppressive treatment should be monitored for HG. Initiation of IgG-RT can be considered depending on the severity of infections, IgG level, and immune function. A regularly scheduled and titrated dosing regimen has shown the best evidence for reducing severe bacterial infections. The IgG trough should be monitored closely and titrating to a biological trough can be considered. Safety labs and side-effects should also be regularly monitored. Various decision and monitoring strategies exist when deciding to discontinue IgG-RT.

Fundamentally, IgG-RT makes physiological sense in MM and has the potential to significantly improve clinical outcomes. Recent studies show emerging benefit and potential for IgG-RT to improve infectious outcomes in the appropriate clinical setting, but yet has not shown an improvement in mortality outcomes. These current findings raise the question of other immunologic mechanisms and treatment effects at play that differ between MM versus primary agammaglobulinemia. However, these findings are also confounded by the heterogeneity of MM treatment profiles that makes standardized prospective clinical trials challenging. Additional confounding factors, such as CVC placement, glucocorticoid administration, and other immunosuppressive regimens, may also increase the risk for infection independently of low IgG. At this time, IgG-RT initiation involves shared decision-making between the clinician and patient, balancing the benefits of reducing bacterial infection with potential risks that may be heightened in hematologic malignancy. We propose a comprehensive algorithm for starting and managing IgG-RT in MM based on the available literature (Fig. 3). Moving forward, many opportunities exist in designing standardized clinical trials that may finally show concrete benefit in a subpopulation of MM that can open the door to understanding mechanisms of other hematologic and immunologic disorders. Review of the literature suggests that our current understanding of HG in MM is fragmented, and a multi-disciplinary collaborative approach with Hematology/Oncology and Allergy/Immunology will significantly progress the field and benefit patients.
